# The diagnostic and prognostic value of antithrombin III activity for sepsis-induced coagulopathy in septic patients: a prospective observational study

**DOI:** 10.3389/fmed.2025.1645146

**Published:** 2025-12-03

**Authors:** Yuting Li, Feng Zhang, Hongxiang Li, Yao Fu, Yumeng Chen, Youquan Wang, Dong Zhang

**Affiliations:** Department of Critical Care Medicine, The First Hospital of Jilin University, Changchun, Jilin, China

**Keywords:** sepsis-induced coagulopathy, sepsis, antithrombin III activity, diagnosis, prognostic assessment

## Abstract

**Background:**

There are currently no suitable biomarkers for early diagnosis and prognostic evaluation of sepsis-induced coagulopathy (*SIC*), therefore, studying the diagnostic and prognostic value of antithrombin III (AT-III) activity in *SIC* may be useful for early identification and intervention of *SIC*.

**Methods:**

This study is a single-center cohort study, prospectively enrolling patients with sepsis admitted to the ICU from March 2023 to March 2024. Based on whether the *SIC* score was greater than or equal to 4, the enrolled sepsis patients were divided into the *SIC* group and the non-*SIC* group. The *SIC* scoring system consists of three parameters: International normalized ratio (INR), platelet count, and Sequential Organ Failure Assessment (SOFA) score. The measurement of AT-III activity was completed within 12 h of the patient being admitted to the ICU. The receiver operating characteristic (ROC) curve analysis and area under the ROC curve (AUC) were used to evaluate the accuracy of different biomarkers in the diagnosis and prognostic assessment of *SIC*. The DeLong Test was employed to compare whether there was a significant difference between AUCs. Kaplan-Meier survival curve was plotted and Log-rank test was performed to compare the 28-day survival rates among different groups.

**Results:**

This study included a total of 366 patients with sepsis, among which 235 (64.2%) were in the *SIC* group and 131 (35.8%) were in the non-*SIC* group. The AT-III activity in the *SIC* group was significantly lower than that in the non-*SIC* group (*P* < 0.001). ROC curve analysis showed that the AUC for AT-III activity was 0.799 (*P* < 0.001), the AUC for platelets was 0.806 (*P* < 0.001), the AUC for Sequential Organ Failure Assessment (SOFA) score was 0.746 (*P* < 0.001), and the AUC for international normalized ratio (INR) was 0.765 (*P* < 0.001). The results of the DeLong Test showed that the AUC for AT-III activity in diagnosing *SIC* had no statistically significant difference compared with the AUCs of the traditional diagnostic indicators, including platelets, SOFA score, and INR (*P* > 0.05). The cut-off value of AT-III activity for diagnosing *SIC* is 59.7%, with a sensitivity of 79.91%, specificity of 69.77%, positive predictive value (PPV) of 82.59%, and negative predictive value (NPV) of 65.94%. There was no statistical difference in AT-III activity between the survival and non-survival groups of *SIC* patients (*P* > 0.05). The proportion of shock and the duration of vasopressor use were both lower in the high AT-III group (≥ 59.7%) than in the low AT-III group < 59.7%) (*P* < 0.05). Kaplan-Meier survival curves showed that there was no statistically significant difference in the 28-day survival probability between the high AT-III group and the low AT-III group (*P* = 0.350).

**Conclusion:**

AT-III activity is a potentially helpful adjunctive biomarker for diagnosing *SIC* that performs similarly to the biomarkers and scores currently used to diagnose *SIC*.

## 1 Background

Sepsis is defined as life-threatening organ dysfunction caused by a dysregulated host response to infection (1). It poses a significant threat to the survival of patients admitted to the intensive care unit (ICU). The incidence and mortality of sepsis remain high, making it one of the leading causes of death in the ICU worldwide (2). The underlying pathophysiological mechanisms of sepsis appear to be complicated and involve disorders in excessive inflammation, immune dysfunction, and multiple organ systems (3, 4). Among all patients with sepsis, those who develop coagulopathy are at higher risk of death (5). Sepsis-induced coagulopathy (*SIC*) is a serious complication of sepsis, often leading to multiple organ dysfunction syndrome (MODS) and poor prognosis (6, 7). The *SIC* criteria and its scoring system were constructed in 2017 to categorize coagulopathy in sepsis (8). Subsequently, the Scientific Standardization Committee (SSC) on Disseminated Intravascular Coagulopathy (DIC) of the International Society on Thrombosis and Haemostasis (ISTH) adopted *SIC* for the diagnosis of early phase DIC in 2019 (9). *SIC* is characterized by a prolonged international normalized ratio (INR) and reduced platelet counts, which can be attributed to the elevated level of tissue factor on the surface of circulating endothelial cells and the impaired balance between anticoagulant and fibrinolytic pathways when exposed to sepsis (10). Activation of the coagulation system and ensuing thrombin generation is dependent on expression of tissue factor and the simultaneous down-regulation of endothelial-bound anticoagulant mechanisms and endogenous fibrinolysis. Early detection of coagulation disorders is crucial for assessing the severity and predicting the prognosis of sepsis (11). Recent studies have demonstrated that inflammation and coagulation collaboratively contribute to the pathogenesis of organ dysfunction (12).

Antithrombin III (AT-III) is a serine protease inhibitor synthesized in the liver. As a natural anticoagulant, AT-III maintains the balance of coagulation and anticoagulation in the body by irreversibly inhibiting the activity of thrombin and other coagulation factors (13). AT-III, as an important physiological anticoagulant protein in the human body, plays a crucial role in maintaining the balance between coagulation and anticoagulation. In addition, AT-III also has an independent antagonistic effect on inflammatory response, which can promote the synthesis and release of anti-inflammatory mediators, inhibit the synthesis of inflammatory mediators, reduce the adhesion between white blood cells and endothelial cells, alleviate vascular damage, and inhibit the activation of pro-inflammatory cytokines (14). In the pathophysiological process of sepsis, the measured activity of AT-III is significantly reduced, and its mechanism includes excessive thrombin production, increased vascular leakage, impaired synthesis ability, and protease degradation (15). A study by Matsubara and colleagues reported that the activity of AT-III may have potential as a unique biomarker for sepsis and sepsis induced DIC (16). The activity changes of AT-III are closely related to the coagulation status and prognosis of sepsis patients (17–19). The measured activity of AT-III is significantly reduced in patients with sepsis and coagulation dysfunction, which not only helps to identify coagulation dysfunction early, but may also serve as an important indicator for predicting patient prognosis.

Despite the deepening understanding of coagulation dysfunction in sepsis, there are currently no suitable biomarkers for early diagnosis and prognostic evaluation of *SIC*. Therefore, studying the diagnostic and prognostic value of AT-III activity in *SIC* may be useful for early identification and intervention of *SIC*. The aim of this study is to explore the value of AT-III activity in diagnosing *SIC* and predicting 28- day mortality in *SIC* patients, in order to provide evidence for early diagnosis and treatment of *SIC*.

## 2 Materials and methods

### 2.1 Study design

This study was a single-center cohort study, prospectively enrolling patients with sepsis admitted to the ICU of a tertiary general hospital (The First Hospital of Jilin University in Changchun, China) from March 2023 to March 2024. Clinical data of septic patients were collected through the electronic medical records system. All participants in this study provided informed consent forms. This study has been approved by the Ethics Committee of the First Hospital of Jilin University (Approval number: 2022-013).

### 2.2 Study population

Adult patients fulfilling the diagnostic criteria for sepsis stated in the third international consensus definitions for sepsis and septic shock (Sepsis-3) (1) were enrolled.

The inclusion criteria were: (1) adult (≥ 18 years old); (2) met the definition of Sepsis 3.0 criteria, which is defined as a suspected infection combined with an acute increase in Sequential Organ Failure Assessment (SOFA) score ≥ 2.

The exclusion criteria were: (1) age < 18 years; (2) patients in the acute phase of trauma or with active bleeding (such as flail chest, obvious contusions of the lungs, liver, spleen, retroperitoneal bleeding, pelvic fractures, gastrointestinal bleeding, etc.); (3) those with a history of congenital bleeding diathesis, such as hemophilia; (4) patients with fulminant hepatitis, decompensated cirrhosis, or other severe liver diseases; (5) patients who had been administered heparin and heparin-like substances (including low molecular weight heparin, dalteparin, etc.) within 12 h before admission to the ICU; (6) patients who were on warfarin and had an INR exceeding the normal range within 7 days before admission to the ICU; (7) patients who had undergone thrombolytic therapy within 3 days before admission; (8) patients who had been on platelet inhibitors (such as aspirin, clopidogrel, tirofiban, dipyridamole, etc.) within 7 days before admission to the ICU; (9) patients who were on other new anticoagulant drugs (Xa factor inhibitors such as apixaban, rivaroxaban, edoxaban, etc., and direct thrombin inhibitors such as dabigatran); (10) patients with thrombotic microangiopathy; (11) patients who did not provide informed consent or withdrew consent during the study. The patients were divided into *SIC* group and non-*SIC* group according to whether the *SIC* score ≥ 4 (8). Two investigators determined eligibility and patient allocation to the two groups based on *SIC* score. The score system for *SIC* is shown in Supplementary Table 1.

### 2.3 Data collection

Within 12 h of ICU admission, patients had the following measured: (1) AT-III activity; (2) creatinine, blood urea nitrogen, total bilirubin, platelets, and lactate to reflect end-organ function; (3) leukocyte count, procalcitonin (PCT), and high-sensitivity C-reactive protein (CRP) to indicate inflammation; and (4) prothrombin time (PT), activated partial thromboplastin time (aPTT), thrombin time (TT), INR, fibrinogen (FBG), D-dimer, and fibrin degradation products (FDP) to reflect coagulation status. In addition, we collected patient demographic information, infection site, underlying diseases, disease severity indicators such as SOFA score, the Acute Physiology and Chronic Health Evaluation (APACHE) II score, and clinical outcomes such as mechanical ventilation duration, ICU length of stay, hospital length of stay, in-hospital mortality, and 28-day mortality. According to the Sepsis-3 criteria, patients with septic shock were identified by a vasopressor requirement to maintain a mean arterial pressure of 65 mmHg or greater and serum lactate level > 2 mmol/L (> 18 mg/dL) in the absence of hypovolemia (1).

The method for measuring AT-III activity was the chromogenic (synthetic substrate) assay. The test was performed on a sample of the patient’s citrated plasma by hospital clinical laboratory. The test measured how well a patient’s AT-III can inhibit a known amount of a clotting enzyme. The amount of uninhibited enzyme left was measured by its ability to cleave a color-producing substrate. The result is reported was a percentage of normal AT-III activity.

### 2.4 Statistical analysis

Continuous variables that follow a normal distribution were expressed as the mean ± standard deviation (SD) and compared using an independent-samples *t*-test. Continuous variables that did not follow a normal distribution were expressed as the median (interquartile range) and compared using the Mann-Whitney U test. The Kolmogorov-Smirnov test was used to assess the normality of data distribution. Categorical variables were presented as numbers and percentages and compared using the chi-squared test. Receiver operating characteristic (ROC) curves were used to analyze the accuracy of AT-III activity in the diagnosis and prognostic evaluation of *SIC* (20), and the Youden index was calculated (21). The optimal threshold for diagnosis and prognostic evaluation was determined when the Youden index was maximized. An area under the ROC curve (AUC) of 70–90% indicated good accuracy in diagnosis and evaluation, the DeLong Test was employed to compare whether there was a significant difference between AUCs. Kaplan-Meier survival curves were plotted and compared using the Log-rank test to assess 28-day survival rates across groups. All comparisons were 2-sided at an alpha level of 0.05, and a *P*-value < 0.05 was considered statistically significant. All statistical analyses were performed using SPSS version 25.0 (IBM, Armonk, NY, United States) and R version 4.2.1 (RStudio, PBC, Boston, MA).

## 3 Results

### 3.1 Comparison of baseline data in *SIC* and non-*SIC* patients

**FIGURE 1:**
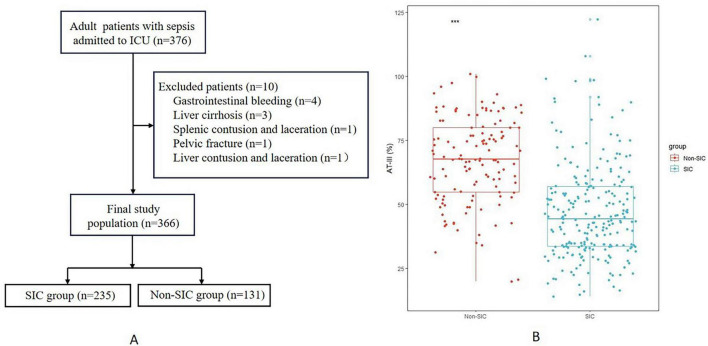
Flowchart for patient inclusion **(A)** and box plot of AT-III activity results **(B)**. ***Represents *P* < 0.001; Mann-Whitney *U*-test was used to compare the levels of AT-III activity between the two groups. AT-III, antithrombin III; ICU, intensive care unit; SIC, sepsis-induced coagulopathy.

This study included a total of 366 patients with sepsis, among which 235 (64.2%) were in the *SIC* group and 131 (35.8%) were in the non-*SIC* group (Figure 1A). There were no statistically significant differences between the two groups in terms of gender, weight, white blood cell count and high-sensitivity C-reactive protein (*P* = 0.186, *P* = 0.941, *P* = 0.103, and *P* = 0.826, respectively). However, SOFA score (*P* < 0.001), INR (*P* < 0.001), procalcitonin (*P* < 0.001), lactic acid (*P* < 0.001), and APACHE III score (*P* = 0.020) were significantly higher in the *SIC* group compared to the non-*SIC* group on ICU admission, while platelet count (*P* < 0.001) was significantly lower, and other indicators (*P* < 0.05) between the two groups at the time of admission, as shown in Table 1.

**TABLE 1:** Baseline data of *SIC* and non-*SIC* patients.

Characteristic	Non-*SIC* group (*n* = 131)	*SIC* group (*n* = 235)	*P*-value
Male	75(57.25)	151 (64.25)	0.186
Age (years)	71.000 (61.00, 74.00)	65.00 (54.00, 73.00)	0.001
Weight (kg)	68.00 (60.00, 70.00)	67.00 (60.00, 75.00)	0.941
SOFA	5.00 (3.00, 7.00)	9.00 (6.00, 11.00)	< 0.001
APACHE II	13.00 (9.00, 16.00)	15.00 (12.00, 18.00)	0.020
**Past medical history**			
Hypertension	40 (30.53)	82 (4.89)	0.396
Diabetes mellitus	20 (15.27)	51 (21.70)	0.136
Coronary artery disease	6 (4.58)	25 (10.63)	0.046
Cancer	7 (5.34)	18 (7.66)	0.380
Cerebral infarction/hemorrhage	13 (9.92)	21 (8.94)	0.755
CRE(μmol/L)	88.70 (58.40, 144.00)	140.10 (85.80, 234.80)	< 0.001
TBIL (μmol/L)	13.50 (9.70, 20.00)	18.00 (12.20, 35.70)	< 0.001
WBC (× 10^9^/L)	12.01 (8.63, 16.48)	11.37 (7.12, 17.37)	0.103
PLT (× 10^9^/L)	218.00 (170.00, 292.00)	120.00 (71.00, 194.00)	< 0.001
PCT (ng/ml)	2.90 (0.48, 14.25)	16.00 (3.35, 70.00)	< 0.001
CRP (mg/L)	170.00 (91.60, 264.73)	158.89 (88.87, 252.17)	0.826
Lactic acid (mmol/L)	1.40 (1.10, 2.00)	2.30 (1.50, 3.50)	< 0.001
PT(s)	12.25 (11.43, 13.70)	14.75 (13.40, 16.70)	< 0.001
aPTT (s)	29.90 (27.60, 32.80)	33.80 (28.90, 41.00)	< 0.001
INR	1.15 (1.05, 1.25)	1.33 (1.18, 1.53)	< 0.001
FBG (g/L)	5.06 (3.86, 6.62)	4.40 (2.65, 6.24)	< 0.001
D-dimer (mg/L)	3.50 (2.01, 7.80)	7.35 (3.50, 13.73)	< 0.001
FDP (μg/mL)	10.57 (6.00, 24.04)	20.50 (10.25, 40.56)	< 0.001
**Site of infection**			
Pulmonary	53 (40.46)	78 (33.19)	0.164
Intra-abdominal	57 (43.51)	107 (45.53)	0.709
Genitourinary	6 (4.58)	26 (11.06)	0.182
Skin/soft tissue infection	11 (8.40)	15 (6.38)	0.472
Blood	4 (3.05)	9 (3.83)	0.700

APACHE II, Acute Physiology and Chronic Health Evaluation II; aPTT, activated partial thromboplastin time; CRP, C-reactive protein; CRE, creatinine; FBG, fibrinogen; FDP, fibrin degradation product; INR, international normalized ratio; PCT, procalcitonin; PLT, platelets; PT, prothrombin time; SOFA, Sequential Organ Failure Assessment; TBIL, total bilirubin; WBC, white blood cells; Continuous variables (non-normal distribution) are expressed as median (interquartile range) and compared using the Mann-Whitney *U*-test. Categorical variables are expressed as number (percentage) and compared using the chi-squared test.

### 3.2 ROC curve analysis for the diagnosis of *SIC* using AT-III activity

**FIGURE 2:**
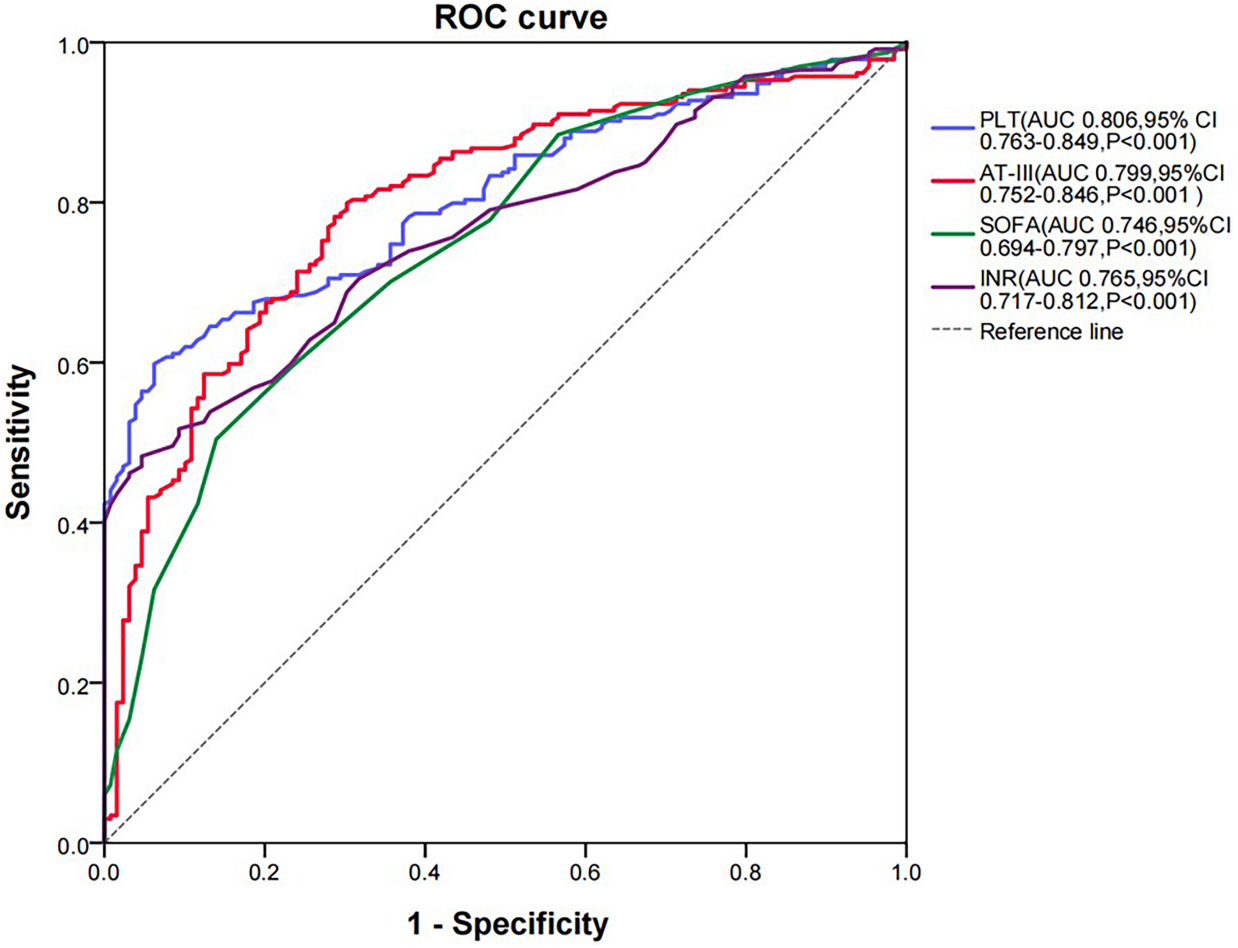
ROC curve analysis of *SIC* diagnosis. AT-III, antithrombin III; AUC, area under the curve; CI, confidence interval; INR, international normalized ratio; PLT, platelet; ROC, receiver operating characteristic.

In this study, the results of AT-III activity in the *SIC* group were significantly lower than those in the non-*SIC* group (44.40 vs. 67.60%, respectively; *P* < 0.001) (Figure 1B). Therefore, we used ROC curve analysis to evaluate the value of AT-III in the diagnosis of *SIC*. The current diagnostic indicators for *SIC* include SOFA score, INR, and platelet count. To evaluate the diagnostic efficacy of AT-III activity, we compared its ROC curve area under the curve (AUC) with that of SOFA score, INR, and platelet count. Since the higher the SOFA score and INR, the greater the possibility of diagnosing *SIC*, which is different from the trend of changes in platelet count and AT-III activity, we performed reciprocal processing on SOFA score and INR in the ROC curve analysis. The results showed that the AUC for AT-III activity was 0.799 (*P* < 0.001), the AUC for platelets was 0.806 (*P* < 0.001), the AUC for SOFA score was 0.746 (*P* < 0.001), and the AUC for INR was 0.765 (*P* < 0.001) (Figure 2). An AUC of 70–90% indicates good accuracy of diagnostic and evaluation results, so AT-III activity has good accuracy in diagnosing *SIC*. The results of the DeLong Test showed that the AUC for AT-III activity in diagnosing *SIC* had no statistically significant difference compared with the AUCs of the traditional diagnostic indicators (8), including platelets, SOFA score, and INR (*P* = 0.928, *P* = 0.104, and *P* = 0.211, respectively) (Supplementary Table 2).

### 3.3 Cut-off value analysis for diagnosing *SIC* using AT-III activity

Our findings suggest that AT-III activity showed a decreasing trend in the *SIC* group. When the Youden index is at its maximum, the activity of AT-III is 59.7%, suggesting that the cut-off value of AT-III activity for diagnosing *SIC* is 59.7%, with a sensitivity of 79.91%, specificity of 69.77%, positive predictive value (PPV) of 82.59%, and negative predictive value (NPV) of 65.94%.

### 3.4 Analysis of AT-III activity in 28-day survival and non-survival groups of *SIC* patients

The age, APACHE II score, and lactate levels of patients with *SIC* were all significantly higher in the 28-day non-survival group compared to the survival group (*P* = 0.029, *P* = 0.004, and *P* = 0.004, respectively). There were no statistically significant differences between the two groups in terms of AT-III activity, SOFA score, INR, and platelet count (*P* = 0.114, *P* = 0.055, *P* = 0.650, and *P* = 0.811, respectively). There was also no statistical difference in AT-III activity between the survival and non-survival groups of *SIC* patients (*P* = 0.114) (Table 2). Therefore, AT-III activity may not serve as a predictive indicator for the 28-day mortality rate of *SIC* patients.

**TABLE 2:** Baseline data of *SIC* patients in the 28-day survival and non-survival groups.

Characteristic	Survival group (*n* = 189)	Non-survival group (*n* = 46)	*P*-value
Male,	126 (66.67)	25 (54.35)	0.118
Age (years)	65.00 (53.00, 72.00)	68.50 (60.50, 74.25)	0.029
Weight (kg)	67.45 ± 0.87	66.48 ± 1.38	0.608
SOFA	8.56 ± 0.29	9.80 ± 0.55	0.055
APACHE II	14.00 (11.00, 18.00)	16.00 (14.00, 20.00)	0.004
CRE (μmol/L)	140.10 (83.30, 232.25)	139.40 (90.98, 252.23)	0.774
TBIL (μmol/L)	18.00 (12.30, 35.70)	19.90 (10.40, 38.25)	0.904
WBC (× 10^9^/L)	13.01 ± 0.67	12.83 ± 1.12	0.904
PLT (× 10^9^/L)	121.00 (72.50, 192.50)	117.50 (67.75, 194.00)	0.811
PCT (ng/mL)	17.00 (4.81, 72.40)	10.95 (1.80, 48.50)	0.190
CRP (mg/L)	179.57 ± 7.91	156.73 ± 16.31	0.204
Lactic acid (mmol/L)	2.20 (1.40, 3.30)	2.95 (1.88, 4.75)	0.004
PT (s)	14.30 (13.30, 16.60)	15.30 (13.55, 17.00)	0.604
aPTT (s)	34.00 (30.00, 40.80)	32.95 (27.98, 43.00)	0.661
INR	1.32 (1.20, 1.53)	1.37 (1.16, 1.58)	0.650
FBG (g/L)	4.87 ± 0.24	4.13 ± 0.33	0.148
D-Dimer (mg/L)	7.62 (3.27, 13.37)	7.06 (3.54, 14.36)	0.901
FDP (μg/mL)	20.95 (9.60, 41.66)	19.00 (13.29, 39.98)	0.677
AT-III activity (%)	45.30 (34.00, 57.10)	38.30 (32.70, 57.40)	0.114

APACHE II, Acute Physiology and Chronic Health Evaluation II; aPTT, activated partial thromboplastin time; AT-III, antithrombin III; CRE, creatinine; CRP, C-reactive protein; FBG, fibrinogen; FDP, fibrin degradation product; INR, international normalized ratio; PCT, procalcitonin; PLT, platelets; PT, prothrombin time; SOFA, Sequential Organ Failure Assessment; TBIL, total bilirubin; WBC, white blood cells; Continuous variables (normal distribution) are expressed as the mean ± standard deviation (SD) and compared using an independent-samples *t*-test. Continuous variables (non-normal distribution) are expressed as the median (interquartile range) and compared using the Mann-Whitney U test. Categorical variables are expressed as number (percentage) and compared using the chi-squared test.

### 3.5 Analysis of clinical outcomes in septic patients with high and low AT-III groups

**FIGURE 3:**
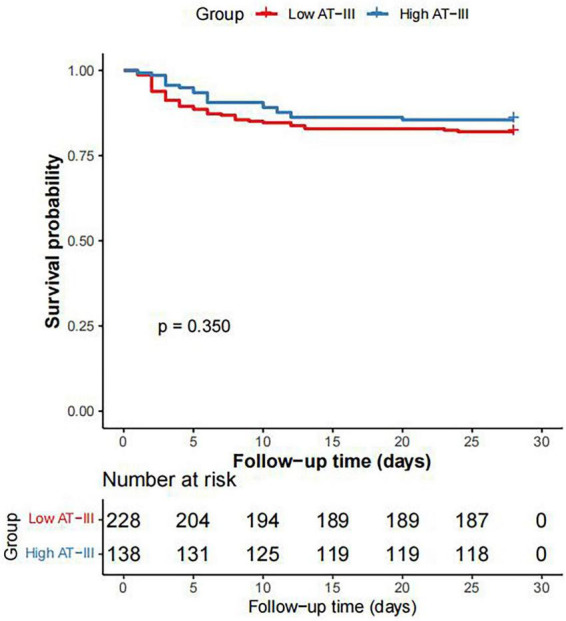
Kaplan-Meier survival curve of 28-day survival rate in patients with sepsis between the high AT-III group and the low AT-III group.

Based on the cut-off value of AT-III activity for diagnosing *SIC*, septic patients were divided into a high AT-III group (AT-III activity ≥ 59.7%) and a low AT-III group (AT-III activity < 59.7%). There were 138 patients in the high AT-III group and 228 patients in the low AT-III group. The proportion of shock and the duration of vasopressor use were both lower in the high AT-III group than in the low AT-III group (*P* < 0.001). There were no statistically significant differences between the two groups in terms of mechanical ventilation time, 28-day survival time, ICU length of stay, hospital length of stay, hospital mortality rate, and 28-day mortality rate (*P* = 0.719, *P* = 0.308, *P* = 0.051, *P* = 0.056, *P* = 0.188, and *P* = 0.338, respectively) (Table 3). Kaplan-Meier survival curves were plotted and Log-rank tests were performed to further clarify the impact of AT-III activity on the 28-day survival rate of septic patients. The results showed that there was no statistically significant difference in the 28-day survival probability between the high AT-III group and the low AT-III group (*P* = 0.350) (Figure 3).

**TABLE 3:** Comparison of clinical outcomes between septic patients in the high AT-III group and the low AT-III group.

Clinical outcomes	High AT-III group (*n* = 138)	Low AT-III group (*n* = 228)	*P*-value
Mechanical ventilation time (h)	73.00 (4.00, 168.50)	68.50 (16.00, 157.00)	0.719
Shock proportion	50 (36.23)	158 (69.30)	< 0.001
Duration of vasopressor use (d)	0 (0, 2.00)	2.00 (0, 5.00)	< 0.001
28-day survival time (d)	28.00 (5.00, 28.00)	28.00 (13.00, 28.00)	0.308
ICU length of stay (d)	6.50 (4.00, 13.25)	6.00 (4.00, 11.00)	0.051
Hospital length of stay (d)	9.00 (6.00, 17.25)	8.00 (5.00, 15.00)	0.056
Hospital mortality	8 (5.80)	13 (5.70)	0.188
28-day mortality	20 (14.49)	41 (17.98)	0.338

AT-III, antithrombin III; ICU, intensive care unit; Continuous variables (non-normal distribution) are expressed as median (interquartile range) and compared using the Mann-Whitney *U*-test. Categorical variables are expressed as number (percentage) and compared using the chi-squared test, the cut-off value of AT-III activity is 59.7%.

## 4 Discussion

This study found that AT-III activity has good accuracy in diagnosing *SIC*, and AT-III activity may be a potential biomarker for the diagnosis of *SIC*. However, AT-III activity is not a significant predictor of mortality in septic patients. AT-III is a 58 kDa plasma glycoprotein synthesized in the liver and belongs to the serine protease inhibitor family. AT-III irreversibly inhibits activated factor X and thrombin in a 1:1 ratio, forming a protease-AT complex. In addition to inhibiting inflammation induced by activated factor X and thrombin, AT-III also participates in the inhibition of vascular endothelial inflammatory responses (22). Sepsis and septic shock often lead to the activation of the coagulation system and the disruption of one of the components of the anticoagulation system, AT-III, resulting in a decrease in AT-III activity.

In our study, we observed a significant reduction in AT-III activity among patients with *SIC*. The mechanism of reduced AT-III activity in *SIC* patients has not been fully elucidated and may be related to the following three potential mechanisms (23). First, since the physiological function of AT-III is to neutralize thrombin and other coagulation factors, AT-III is consumed in patients with activated coagulation (24). Second, during sepsis, the synthesis of AT-III in the liver is inhibited, and AT-III activity gradually decreases with the reduced production of AT-III (25). Third, due to the endothelial damage in sepsis leading to increased capillary permeability, circulating AT-III can rapidly leak from the intravascular to the extravascular space. These factors together lead to the reduced AT-III activity in *SIC* patients (26).

AT-III activity is an important physiological anticoagulant, capable of inhibiting about 80% of the coagulant activity of thrombin and coagulation factors VII, IX, X, XI, and XII. We believe that the reduction in AT-III activity is a critical factor contributing to the worsening of coagulation function in sepsis patients. Specifically, in coagulation dysfunction caused by sepsis, AT-III activity usually decreases and is associated with disease severity and high mortality. The reduction of AT-III activity leads to a decrease in the ability to inactivate thrombin, resulting in further deterioration of coagulation function and even the occurrence of multiple organ dysfunction. Iba et al. (27) reported that in infected patients without organ dysfunction, AT-III activity dropped to about 80% of the normal level, while in sepsis patients with organ dysfunction, it dropped to about 60%, and in sepsis patients with DIC, it dropped to about 40%. In our cohort, we established a threshold of 59.7% for AT-III activity to diagnose *SIC*. This threshold reflects the progressive decline in AT-III activity as organ dysfunction and coagulation dysfunction advance in sepsis. Our results suggest that monitoring AT-III activity could serve as a valuable tool for early identification and management of *SIC*, potentially improving clinical outcomes by guiding timely interventions.

Our study results not only validate the findings of previous studies but also provide more specific evidence for the application of AT treatment in patients with sepsis-induced DIC. A nationwide multicenter retrospective observational study showed that patients with sepsis-induced DIC and very low AT-III activity (≤ 43%) are the best indication for AT treatment (28). In addition, no increase in bleeding complications due to AT treatment was found in this patient subgroup. In previous clinical trials, even septic patients with AT-III activity of 70% were approved to start AT treatment. The results of these trials did not clarify the threshold of AT activity at the onset of treatment for sepsis-induced DIC, but these thresholds are likely based on the normal range of AT activity (70–120%) (29). The *post hoc* analysis of the KyberSept study showed that patients with sepsis-induced DIC were more likely to have AT-III activity < 60% than non-sepsis-induced DIC patients (72 vs. 15%) (30). Moreover, AT treatment can significantly improve the survival rate of patients with sepsis-induced DIC (31), and AT treatment may be more effective for patients with lower AT-III activity.

In our study, we further explored the pathophysiological mechanisms of *SIC* and the value of its diagnostic markers. *SIC* is considered to be caused by the activation of systemic intravascular coagulation and microvascular endothelial injury, leading to extensive microvascular thrombosis and organ failure. Many studies have shown that the coagulation dysfunction in sepsis is associated with organ failure and is a risk factor for clinical death (32, 33). Therefore, in order to accurately assess the coagulation function in sepsis, various coagulation markers are clinically measured in sepsis patients, such as fibrin/fibrinogen degradation products (FDP), prothrombin time, platelet count, fibrinogen, INR, and AT-III activity. However, in the complex pathophysiology of sepsis and its accompanying coagulation dysfunction, traditional coagulation markers cannot reflect the full picture of coagulation function and it is also difficult to verify the validity of the markers. Therefore, the clinical significance and effectiveness of various coagulation indicators for the treatment of sepsis are still unclear. Although the results of our study showed no statistical difference in AT-III activity between the 28-day survival group and the death group of *SIC* patients, and AT-III activity cannot be used as a predictive indicator of 28-day mortality in *SIC* patients, as a diagnostic indicator for *SIC*, AT-III has a good performance in both sensitivity and specificity for the diagnosis of *SIC*. In addition, the results of our study showed that the proportion of shock and the duration of vasopressor use in the high AT-III group (AT-III activity ≥ 59.7%) were lower than those in the low AT-III group (AT-III activity < 59.7%), suggesting that there is a close relationship between decreased AT-III activity and the occurrence of shock in sepsis patients.

In our study, we further validated the significance of AT-III activity detection in the diagnosis of *SIC* and proposed new insights based on our research findings. On the one hand, abnormal levels of AT-III can serve as an important diagnostic basis for *SIC*. When the level of AT-III is too low, it may indicate that the body is more prone to forming uncontrollable thrombi, which is consistent with the pathological process of *SIC*. On the other hand, our study also explored the potential role of AT-III activity in guiding clinical treatment. We found that by monitoring changes in AT-III activity, we can more accurately assess the coagulation status of patients, thereby guiding anticoagulant therapy. For instance, heparin can increase the activity of AT-III by 1,000 times through competitive binding to AT-III and preventing its interaction with heparan sulfate on endothelial cells (34). Since the anticoagulant effect of heparin depends on the activity of AT-III, monitoring the changes in AT-III levels can also assess the coagulation status of patients, guide clinical anticoagulant therapy, and thereby improve the prognosis of patients. In the future, with the continuous progress of medical technology and in-depth research on AT-III, its role in the diagnosis, treatment, and prognostic assessment of *SIC* will become clearer and more important. Based on our research findings, we believe that developing more sensitive and specific AT-III detection technologies can detect coagulation abnormalities earlier, providing strong support for clinical intervention. At the same time, establishing a comprehensive diagnostic model by combining other biomarkers and clinical information will further improve the diagnostic accuracy and therapeutic efficiency of *SIC*. In summary, as an indicator that is easy to detect, readily available, and highly universal, AT-III activity has broad application prospects and important clinical value in the diagnosis of *SIC*. In the future, with ongoing in-depth research and technological development, AT-III activity is expected to play a more important role in the diagnosis and treatment of *SIC*.

There are several limitations in our study. First, since it is a single-center study, the sample size is relatively small. Second, the dynamic changes of AT-III activity during the sepsis process were not continuously monitored. Third, due to the single-center nature and small heterogeneous patient population, the generalizability of our results is limited. Therefore, larger-scale and multicenter research is still required in the future.

## 5 Conclusion

AT-III activity decreases during *SIC*, and AT-III activity is a potential biomarker for diagnosing *SIC* with diagnostic efficacy similar to current indicators of SOFA score, INR and platelets.

## Data Availability

The raw data supporting the conclusions of this article will be made available by the authors, without undue reservation.
