# Supplementary material for: The diagnostic and prognostic value of antithrombin III activity for sepsis-induced coagulopathy in septic patients: a prospective observational study
Source: Front Med (Lausanne). 2025 Dec 3;12:1645146. doi: 10.3389/fmed.2025.1645146 (PMC12708531; doi:10.3389/fmed.2025.1645146)
Supplement: Supplementary file 1 [file Table_1.DOCX]

Supplementary Table1 Score system for sepsis-induced coagulopathy.

| **Category** | **Parameter** | **0 point** | **1 point** | **2 points** |
| --- | --- | --- | --- | --- |
| Prothrombin time | INR | ≤1.2 | >1.2 | >1.4 |
| Coagulation | Platelet count (× 10^9^/L) | ≥150 | <150 | <100 |
| Total SOFA | SOFA four items | 0 | 1 | ≥2 |

INR:international normalized ratio; SOFA: Sequential Organ Failure Assessment; Total SOFA is the sum of the four items (respiratory SOFA, cardiovascular SOFA, hepatic SOFA, renal SOFA), SOFA score was calculated post-hoc by investigators.
