# Supplementary material for: The diagnostic and prognostic value of antithrombin III activity for sepsis-induced coagulopathy in septic patients: a prospective observational study
Source: Front Med (Lausanne). 2025 Dec 3;12:1645146. doi: 10.3389/fmed.2025.1645146 (PMC12708531; doi:10.3389/fmed.2025.1645146)
Supplement: Supplementary file 2 [file Table_2.DOCX]

Supplementary Table 2 Delong test results of AUC for different diagnostic markers

| **Comparison of markers** | **AUC difference** | **95% CI** | **Z value** | **P value** |
| --- | --- | --- | --- | --- |
| AT-III activity vs Platelets | 0.007 | -0.065~0.059 | -0.090 | 0.928 |
| AT-III activity vs SOFA | 0.053 | -0.011~0.117 | 1.628 | 0.104 |
| AT-III activity vs INR | 0.034 | -0.019~0.087 | 1.252 | 0.211 |

AT-III: antithrombin III; AUC: area under the curve; CI: confidence interval; INR: international normalized ratio; SOFA: Sequential Organ Failure Assessment.
